# Progression to Metastasis of Solid Cancer

**DOI:** 10.3390/cancers13040717

**Published:** 2021-02-10

**Authors:** Eldad Zacksenhaus, Sean E. Egan

**Affiliations:** 1 Department of Medicine, Laboratory Medicine & Pathobiology and Medical Biophysics, University of Toronto, Toronto, ON M5G 1L7, Canada; 2 Toronto General Research Institute, University Health Network, Toronto, ON M5G 1L7, Canada; 3 Department of Molecular Genetics, University of Toronto, Toronto, ON M5G 0A4, Canada; 4 Program in Cell Biology, The Peter Gilgan Center for Research and Learning, The Hospital for Sick Children, Toronto, ON M5G 0A4, Canada

Metastatic dissemination of cancer cells, their colonization at distal sites, and ultimate disruption of tissue physiology are the root causes of most deaths from solid cancers, particularly in tumor types where the primary lesion can be easily dissected and discarded [1]. However, most therapies are traditionally based on the biology, driver mutations and/or drug sensitivity, of primary tumor cells. This “under the streetlight” approach, historically justified by a scarcity of metastatic samples, dated technologies, and the assumption that tumors are the same “here, there and everywhere” is being gradually replaced with high resolution analysis of metastases and their precursors using advanced technologies adapted to minute biopsies as well as with new animal models of metastatic cancer that faithfully recapitulate the human disease. Although precision medicine designed for metastases is feasible, the overarching goal of metastatic research is to better understand the dissemination process that leads to macrometastases, identify unique vulnerabilities, and advise appropriate therapeutic interventions to prevent recurrence.

## 1. Progression to Metastasis

From landmark papers in the early 1980s, the role of cooperation between oncogenic mutations in tumor progression has been well defined [2,3]. Indeed, specific programs induced in response to genetic interaction between certain oncogenic drivers are themselves responsible for invasive phenotypes [4]. From analysis of colorectal tumors at different stages, a model was developed to explain oncogenic driver cooperation and the role of individual mutations in progression towards more invasive and lethal diseases [5]. Functional genomic screens have also helped define specific driver cooperation and its role in tumor initiation and progression [6,7]. A common finding from comparative analyses of primary tumors versus metastases is that oncogenic profiles in the two compartments are similar but not identical (e.g., [8,9,10,11]). What is the basis for this incomplete overlap? This question was asked by Bernards and Weinberg, albeit in a different way, nearly 20 years ago; Do oncogenic alterations found in primary tumors suffice to drive metastatic spread, or are additional, metastatic-specific alterations needed? [12]. The former view contends that oncogenic mutations selected by Darwinian competition within a primary tumor enables the emergence of aggressive clones that successfully compete locally and are also equipped with attributes required for metastasis. This idea is supported by the ability of prognostic signatures derived from primary tumors to successfully, though not fully, predict clinical outcomes (e.g., [13]) and the detection of disseminating tumor cells at very early stages of cancer progression [14], indicating that oncogenes and tumor suppressors, which drive early lesions, are suffice to confer metastatic potential. Indeed, *Ras* mutant alleles can promote primary tumor growth and also metastasis [15]. In this regard, *RAS* pathway activation is observed in basal but not luminal breast cancer subtypes [16], and luminal A breast cancer patients with high *RAS* pathway activity exhibit exceedingly poor prognosis [17]. *RAS* pathway activation can occur at presentation, in which case, gene expression-based prognostication identifies high-risk patients (as in basal BC), or it can happen through mutations induced post dissemination/treatment [18]. Indeed, substantial evidence points to the existence of metastatic-specific genes that facilitate dissemination or drug resistance (see references above). For example, alterations in targets of therapy, such as *ESR1*, are almost exclusively selected for in therapy resistant and metastatic recurrent disease [19]. Moreover, whole genome sequencing of colorectal cancer revealed that 19% of mutations are metastatic-specific [20]. Among the metastatic-specific genes is *BRCA2*, a tumor suppressor whose germline loss predisposes to certain types of cancer, indicating that metastatic-specific mutations can play different roles in different contexts (i.e., acting early in breast cancer initiation, but late in CRC progression to metastasis). 

Functional analysis in mice supports the existence of metastatic-specific or metastatic-promoting genes. For example, while high *Akt1* expression accelerates mammary tumor development, *Akt2* promotes metastasis but not primary tumor formation in transgenic mice [21]. Loss of RhoC GTPases does not affect tumor initiation but decreases motility, tumor cell survival and dissemination in MMTV-*PyMT* mice [22]. Also, *Stat3* deletion does not affect primary tumor formation but reduces metastasis by promoting an immunosuppressive microenvironment [23,24]. 

**Figure 1:**
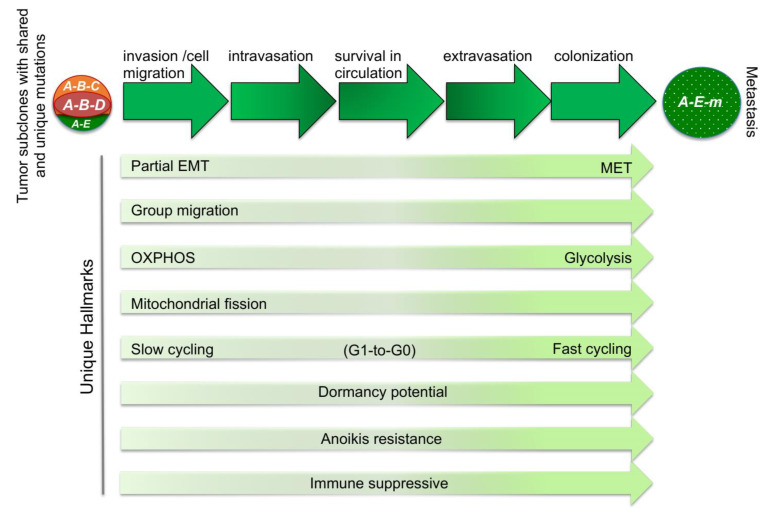
Unique hallmarks of the metastatic cascade. Primary tumors are highly heterogeneous due to clonal evolution and local competition. Dominant subclones with mutations (*A-B-C* or *A-B-D*) may not necessarily sprout metastases. In the depicted scenario, a smaller clone, *A-E*, has acquired an additional mutation, designated *m* (*A-E-m*) at the primary site or post-dissemination that promotes metastatic disease or drug-resistance. Steps in the metastatic cascade and unique hallmarks that may be therapeutically targeted are indicated. A major theme is the plasticity of disseminating tumor cells that enables progression through the “long and winding road” of the metastatic cascade (see text for details).

Metastatic-specific driver mutations could confer a minor selective advantage at the primary site (and therefore would be found in small subclones only detectable by deep sequencing), could be rare (and hence would not accumulate at the primary site) but effective in promoting dissemination, or may actually be acquired at distal sites after tumor cells disseminate. Here, it is important to note that certain alterations that destabilize the genome such as *p53* loss, acquisition of BRCAness, chromothripsis or high-level expression of mutator genes such as cytidine deaminase *APOBEC3A/B*, can increase mutation burden and promote not only primary tumor growth but also tumor cell diversification with the generation of metastatic variants [25,26,27,28,29]. Thus, a simple model to explain these observations involves the development of heterogeneous primary tumors, comprising multiple subclones; the major dominant subclones are driven by oncogenic alterations that select for local growth but not necessarily for metastasis. Then, smaller subclones branch off from dominant subclones while acquiring mutations that empower them to leave the primary niche and disseminate, successfully seeding macrometastases at distal sites. Additional mutations may represent minute subclones in the primary site or occur post-dissemination. In this model, metastasis specific mutations (denoted “*m*” in Figure 1) may reflect parallel evolution post dispersion, passenger mutations, drug resistance, or alterations that directly promote metastasis [30,31]. Regardless of the mechanism, if metastases are driven by and depend on metastasis-specific alterations, then targeting such alterations could well be therapeutic.

## 2. The Metastatic Cascade, Plasticity, and Vulnerabilities

Cancer cells spread through invasion and metastasis. The former involves spread from within a lesion to surrounding local tissues. In principle, a surgeon can remove a locally invasive lesion, however, highly invasive tumors can cause severe morbidity and even mortality. For example, some brain tumors can kill patients even without metastasizing since they cannot be removed without compromising essential brain function. Other solid tumors, including lobular breast cancer can be highly invasive with evidence for local recurrence and bilateral disease. Such tumors can require extensive surgical management through time. Metastasis, by contrast, involves discontiguous dissemination, whereby tumor cells travel through the circulation to implant and grow at secondary tissue sites. This process involves the following distinct steps [32,33], all while avoiding immune surveillance: (i) local invasion and cell migration, (ii) intravasation into the lymphatic or hematogenous system, (iii) survival in the vascular system, (iv) extravasation from vasculature to distal tissue, and (v) colonization (Figure 1). Each of these steps in the metastatic cascade involves unique hallmarks, including complex cellular and metabolic plasticity that can be exploited therapeutically [34,35,36]. For instance, cell migration is facilitated by epithelial-to-mesenchymal transition (EMT), which requires a high degree of plasticity as colonization at distal sites involves the opposite process, mesenchymal-to-epithelial conversion (MET) [37,38]. Induction of N-cadherin but not vimentin has been shown to be required for EMT and metastasis in the lung of MMTV-*PyMT* mice; a recent *N-cadherin* reporter mouse was used to trace EMT and provided the means to dissect this process in vivo [39]. 

While glycolysis promotes anabolic metabolism and rapid cell proliferation, and is selected for during clonal evolution in primary cancers, oxidative phosphorylation (OXPHOS) is observed in circulating tumor cells (CTCs), which revert back to glycolysis during rapid outgrowth of metastases [40]. Whether CTCs arise from glycolytic tumor cells that revert to OXPHOS or from tumor subclones that maintain OXPHOS, remains to be determined. OXPHOS is critical for cell motility and migration [40,41,42]. Indeed, ”slow cycling cells” identified by dye-retention analysis exhibit OXPHOS, increased dissemination, and are associated with tumor relapse, whereas ”fast cycling cells” are glycolytic and less metastatic [43]. The OXPHOS—migration connection may relate to the observation that migratory potential of tumorigenic cells correlates with mitochondrial fission (fragmentation), while less migratory tumor cells harbor fused (concatemeric) forms of this organelle. Mitochondrial fission, which is coordinated by MFN1 and DRP1, promotes mitochondrial distribution to lamellipodia [42,44]. Thus, mitochondrial fission plus a switch to OXPHOS may provide the high local ATP production required for lamellipodia function and migration. High OXPHOS is also a characteristic feature of cancer stem cells (CSCs) with their unique drug responsiveness [35,45]. 

It is commonly thought that CTC maintenance of high OXPHOS, low proliferative state, and dormancy shield them from cytotoxic drugs which act on highly dividing cells. Of interest, a recent cell tagging analysis suggests that both slow and fast proliferating cells can enter a state of dormancy, and then re-enter the cell cycle, re-establishing a CSC hierarchy [46]. Implicit in the aforementioned cell plasticity phenotype is the ability of disseminating tumor cells (DTCs) to exit and then re-enter the cell cycle. While the proclivity of DTCs to enter dormancy protects them from certain therapies, it may expose them to other therapies that block cell cycle re-entry or the OXPHOS-to-glycolysis switch, possibly as part of a larger intervention scheme, which also targets proliferating tumor cells. In addition to targeted therapy, a new therapy, based on low-intensity, intermediate-frequency electric fields, has proven to be highly effective in suppressing cell division in mouse models and recurrent glioblastoma patients, and may be adapted to metastatic disease [47,48]. 

## 3. Plasticity and Hybrid Programs

The section above describes several programs through which tumor cells transition back and forth during metastasis, i.e., EMT versus MET, glycolysis versus OXPHOS, mitochondrial fusion versus fission, CSC/self-renewal versus proliferation/aberrant differentiation, and cell cycling versus dormancy. Tumor cells that are locked into such biological states (e.g., glycolysis) may dominate the primary tumor as they promote rapid proliferation but may fail to sprout lethal metastases. Indeed, in many solid tumors, some tumor cells undergo complete EMT, whereas others only undergo partial EMT, expressing both epithelial and mesenchymal markers; complete EMT leads to single cell migration but no colonization, whereas partial EMT leads to group/collective dissemination and colonization [49,50]. The partial or hybrid EMT state has recently been shown to involve *FAT1* loss, which activates the EMT inducer ZEB1 and the epithelial inducer SOX2, thereby promoting stemness and metastasis [51]. Thus, tumor cells that express mixed phenotypes of partial EMT, elevated glycolysis and OXPHOS, CSC and partial proliferation/aberrant differentiation, and slow cycling may be uniquely capable of disseminating and forming large metastases. 

The aforementioned biological states are interconnected and regulated by oncogenes and tumor suppressors selected for in the course of tumor evolution. For example, multiple oncogenic alterations such as p53 loss and PI3K pathway activation drive glycolysis [52,53]. However, other oncogenic alterations such as *RB1* loss, oncogenic *FER*, and high *SIRT6* expression promote mitochondrial activity and OXPHOS [35,54,55,56,57,58]. Thus, mixed hybrid programs may be achieved by the right balance of specific oncogenic mutations that promote opposing programs. While transitions from one complete program to another may require new genetic alterations, the plasticity associated with partial program acquisition is more subtle and may be readily amenable to post-translational/epigenetic changes in response to stochastic processes or extracellular signaling. In this regard, a major driver of OXPHOS is PGC-1alpha, which is not oncogenically altered in cancer, but its expression level controls mitochondrial biogenesis [40]. Likewise, the MET receptor tyrosine kinase, a major driver of cell motility and invasion, is regulated post-translationally at multiple levels, including degradation by selective autophagy, which is itself affected by oxygen availability and the tumor suppressor VHL [59]. 

## 4. The Tumor Microenvironment

The metastatic cascade involves cell autonomous and non-cell autonomous processes. In fact, the tumor microenvironment (TME), and in particular, the tumor immune microenvironment (TIME), play both positive and negative roles in this process, and are clearly distinct in the primary versus metastatic niches. For example, RNA-seq analysis identified high expression of the *CCL2* chemokine in metastatic medulloblastoma; overexpression of *CCL2* or its receptor, *CCR2*, sufficed to drive hematogenous dissemination in vivo [60]. As noted, although DTCs can migrate as single cells, collective migration is a more efficient route for metastasis [61]. Tumor-associated neutrophils and macrophages (TAMs), as well as myeloid-derived suppressor cells (MDSCs), promote collective migration and intravasation of tumor cells [33,62,63]. Under the influence of colony-stimulating factor 1 (CSF1), TAMs also promote angiogenesis. Extravasation is facilitated by metastasis-associated macrophages (MAM), which secrete VEGFA and further protect DTC. Regulatory T cells (Treg) are instrumental in suppressing tumor surveillance by natural killer cells and cytotoxic CD8+ T cells. Thus, early and late events in the metastatic cascade are dependent on different interactions with the TME. 

Escape from immune surveillance is not only critical for successful dissemination but also for metastatic growth and colonization. Metastatic tumor cells hijack various processes involved in normal homeostasis to escape immune detection and destruction. For example, TGF-β signaling from tumor-associated fibroblasts trap cytotoxic T cells in the stroma, excluding them from tumor parenchyma; inhibition of TGFβ facilitates cytotoxic T-cell response that prevents metastasis [64]. Another mechanism by which tumors evade the immune system involves suppression of cytotoxic T cells through expression of programmed death ligand 1 (PD-L1). PD-L1 binds programmed cell death protein 1 (PD-1) on the surface of cytotoxic CD8+ T cells and attenuate their function [65,66]. Whereas tumor cells are genetically unstable, allowing rare variants to escape therapy, the TME including tumor vasculature and myriad immune cell types comprise genetically stable cells that are more amenable to therapeutic targeting. Indeed, recent success with combination therapies of advanced/metastatic cancer using checkpoint control inhibitors such as anti-PD-1 and anti-PD-L1 antibodies together with therapies targeting the cancer cell compartment is inspiring [67]. Similar therapies combining checkpoint control inhibitors with other vulnerabilities in the TME or the metastatic cascade may offer even better outcomes. The generation of immune-competent mouse models that develop macroscopic metastases (e.g., [68,69]), provide powerful platforms to study metastasis and assess potential new drug combinations that target both the tumor and TME [70]. 

## 5. Metastasis-Based Therapy

With sequence analysis of biopsies taken from metastatic patients, identification of metastasis-specific alterations and design of metastasis-tailored precision medicine, metastasis-based therapy has become technically feasible. A major challenge, however, involves the diverse set of mutations in different metastases from each individual patient [10], as well as the difficulty of obtaining biopsies from certain metastatic sites. Advances in single-cell sequencing technologies, high-resolution immune-landscape analysis, and metabolic profiling of primary tumors, DTCs, and metastatic biopsies, in combination with functional genomic editing of immune-competent mouse models, may help to uncover druggable vulnerabilities that suppress or at least contain the disease before frank metastases develop, without compromising overall survival and well-being.
